# Rational use of SARS-CoV-2 polymerase chain reaction tests within institutions caring for the vulnerable

**DOI:** 10.12688/f1000research.24872.1

**Published:** 2020-07-02

**Authors:** Tom A. Yates, Graham S. Cooke, Peter MacPherson

**Affiliations:** 1 Department of Infectious Disease, Faculty of Medicine, Imperial College London, St Mary's Campus, Norfolk Place, London, W2 1PG, UK; 2 Malawi-Liverpool-Wellcome Trust Clinical Research Programme, Queen Elizabeth Central Hospital, Blantyre, Malawi; 3 Department of Clinical Sciences, Liverpool School of Tropical Medicine, Pembroke Place, Liverpool, L3 5QA, UK; 4 Clinical Research Department, London School of Hygiene & Tropical Medicine, Keppel Street, London, WC1E 7HT, UK

**Keywords:** COVID-19, SARS Coronavirus 2, Infection Prevention and Control, Clinical Diagnostics, Epidemiology

## Abstract

Institutions such as hospitals and nursing or long-stay residential homes accommodate individuals at considerable risk of mortality should they acquire SARS-CoV-2 infection. In these settings, polymerase chain reaction tests play a central role in infection prevention and control. Here, we argue that both false negative and false positive tests are possible and that careful consideration of the prior probability of infection and of test characteristics are needed to prevent harm. We outline evidence suggesting that regular systematic testing of asymptomatic and pre-symptomatic individuals could play an important role in reducing transmission of SARS-CoV-2 within institutions. We discuss how such a programme might be organised, arguing that frequent testing and rapid reporting of results are particularly important. We highlight studies demonstrating that polymerase chain reaction testing of pooled samples can be undertaken with acceptable loss of sensitivity, and advocate such an approach where test capacity is limited. We provide an approach to calculating the most efficient pool size. Given the current limitations of tests for SARS-CoV-2 infection, physical distancing and meticulous infection prevention and control will remain essential in institutions caring for vulnerable people.

## Introduction

Whilst there are significant limitations to the prognostic models that have been published to date ^1^, the association between older age and death in SARS-CoV-2 infection is both clear and striking – the infection fatality rate for people in their 80’s has been estimated at 7.8% ^2^. Infection prevention and control (IPC) is therefore crucial within institutions that bring together many such individuals, such as hospitals and nursing or long-stay residential homes.

Here we discuss the use of polymerase chain reaction (PCR) tests within institutions caring for vulnerable individuals. We emphasise that consideration of test characteristics and the prior probability of SARS-CoV-2 infection is essential in making safe decisions based on PCR results. For example, decisions regarding the allocation of side rooms, whether patients can be cohorted in COVID or non-COVID spaces, and whether staff should be at work. We discuss the different uses of PCR tests, and consider which testing strategies should be prioritised. We explore how pooling samples prior to testing can result in a more efficient use of test capacity, and provide an optimised approach to selecting the number of samples to pool. Finally, we suggest how staff testing programmes for institutional care settings might be efficiently organised. We do not discuss disease surveillance or the role of testing for disease control in the wider community, but would point readers to recent modelling studies ^3, 4^. We only briefly discuss serological tests, as these are not yet in routine use. Our intended audience are those designing testing strategies in health and social care settings, as well as individuals in these settings acting on test results.

## Epidemiology and importance of transmission dynamics for institutional care settings

High rates of SARS-CoV-2 infection with consequent morbidity and mortality have been observed in healthcare facilities and social care settings. Two infection prevalence surveys conducted in April and May 2020 in England found 1.33% and 1.73% of people working in ‘patient-facing healthcare or resident-facing social care roles’ had positive SARS-CoV-2 PCRs as compared with 0.22% and 0.38% of people not working in such roles ^5, 6^. Over much of this period, health and social care workers were one of the few groups permitted to leave their homes to work.

A transmission model parameterised to a typical hospital in the United Kingdom suggests that up to 20% of infections in patients and 89% of infections in healthcare workers may be a result of nosocomial transmission ^7^. Consistent with that, SARS-CoV-2 infections in healthcare workers and staff have been observed to cluster on particular hospital wards ^8^ and a large proportion of hospital staff working in particularly exposed roles, such as housekeepers (34.5%) and those working in acute medicine (33.3%), show serological evidence of having been infected during the initial weeks of the pandemic ^9^.

Large transmission clusters of SARS-CoV-2 infections have been seen in hospitals and elderly care facilities, along with worker dormitories and ships ^10^. In one detailed case report incorporating phylogenetic analysis, a single introduction of SARS-CoV-2 into a private hospital in South Africa led to 80 staff members and 39 patients becoming infected, with 15 patient deaths ^11^. The consequences of transmission within the social care sector have, perhaps, been even more severe. In one well-described outbreak in a ‘skilled nursing facility’, 57/89 (64%) of residents were infected, of whom fifteen died; a case fatality rate of 26% ^12^. Viral sequence data were consistent with two separate introductions of SARS-CoV-2 into the facility ^12^.

A key feature of the epidemiology of SARS-CoV-2 transmission is that viral shedding begins 2–3 days prior to symptom onset ^13^. A high proportion of transmission is thought to occur prior to symptom onset, estimated at 40–50% in studies from different settings ^13– 16^. Disease control strategies that rely on isolation once symptoms develop are, therefore, unlikely to be sufficient ^17^.

Whilst a majority of those with SARS-CoV-2 infection do eventually develop symptoms, an appreciable prevalence of asymptomatic – rather than pre-symptomatic – infection has been described in hospital patients (12.4%) ^18^, nursing home residents (3.9%) ^12^ and healthcare workers (0.5%) ^8^. The contribution that these individuals make to transmission is less clear. The absence of cough and coryza might limit infectivity. Alternatively, a period of viral shedding without the social distancing and reductions in numbers of close contacts, which would have otherwise been prompted by symptom onset, might result in a greater number of secondary infections. Data suggest that pre-symptomatic transmission probably plays a more important role than asymptomatic transmission ^16^. However, until this is better understood it would be prudent to consider both asymptomatic and pre-symptomatic people with positive SARS-CoV-2 PCRs infectious, particularly given this group are initially clinically indistinguishable.

Clearly, the prevalence or prior probability of SARS-CoV-2 infection, among both individuals with and without symptoms, will vary over the course of the pandemic ^19^ and with changes in control strategies.

## Test characteristics

Viral load in the upper airway, the site most readily sampled, is thought to peak around the time of symptom onset ^13^ and to be correlated with symptom severity ^20^. An early analysis suggested that the sensitivity of PCR-based tests decreases with time since symptom onset, with only 67% of nasal and 47% of throat specimens expected to be PCR positive ten days after symptom onset ^21^. In a small series of well-characterised patients with mild disease, test sensitivity was reported to be only 40% five days after symptom onset ^22^. Later analysis, incorporating some of the same data, estimated that the sensitivity of the test was 62% on the day of symptom onset, 80% on day three after symptom onset, then declining to 33% by day 16 post symptom onset ^23^. The sensitivity of a SARS-CoV-2 PCR on the day prior to symptom onset was estimated to be between 6% and 73%, with the imprecision in the estimate reflecting the limited data available on pre-symptomatic patients ^23^. Most of these studies did serial testing and required at least one positive PCR to consider a case confirmed – as a result they may have overestimated the sensitivity of the test. Some of the studies included in Kucirka *et al.* ^23^ allowed probable cases, defined on clinical grounds, with a majority of these probable cases also having IgM or IgG antibodies against SARS-CoV-2.

Data on test specificity is less readily available. As well as the inherent performance of the test, specificity can be impacted by: clinicians swabbing the wrong patient or placing the wrong sticker on the specimen; RNA contamination at any stage in the process; and transcription errors when reporting the result. The extent to which these errors occur will be context specific and dependent on how robust local procedures are. Importantly, PCR based testing only gives a snapshot at one point in time. Individuals early in their infection may test negative then positive a few hours later ^23^. As with all PCR based tests, poor sampling may result in false negatives.

Readily available formulae ^24^ enable the probability of both false negative and false positive tests to be estimated under a range of assumptions about sensitivity, specificity, and the prevalence of the condition in the population being tested.

## Testing symptomatic staff in institutional care settings

A major concern during the COVID-19 pandemic has been health systems’ ability to cope with large numbers of acutely unwell individuals presenting to health facilities within a short period of time, overwhelming capacity. The availability of skilled clinicians is a major constraint on health system capacity and high rates of absenteeism have been reported at many centres ^25^. Absent staff were either symptomatic, self isolating because a household contact was symptomatic, or not working as a result of medical conditions that would put them at risk of severe disease were they to acquire SARS-CoV-2. There has, thus, been great interest in SARS-CoV-2 PCR tests for symptomatic health and social care workers and their symptomatic household contacts. 

Whilst reducing absenteeism is clearly important, a key concern when testing symptomatic individuals is ensuring that false negative results don’t result in avoidable transmission of the virus among keyworkers and the vulnerable individuals they work with. Estimated false negative rates, under a range of plausible assumptions, are presented in Table 1.

**Table 1.** Estimated probability of a false negative SARS-CoV-2 PCR test under various assumptions about the true prevalence of infection and the sensitivity of the test ^24^. All estimates assume a test specificity of 99.5%.

True prevalence of infection	Probability of SARS-CoV-2 infection, given negative PCR test		
	95% test sensitivity	70% test sensitivity	40% test sensitivity
90%	31%	73%	84%
30%	2.1%	11%	21%
5%	0.26%	1.6%	3.1%

As others have also noted ^21, 23, 26^ high rates of false negative tests are seen when the prevalence of infection is high, and when the sensitivity of the test is low. The proportion of health and social care workers who have positive test results should be monitored with these data, ideally, disaggregated by symptoms. Where this proportion is high, there will be an unacceptable false negative rate and institutions should consider asking symptomatic staff to self isolate regardless of their test result. Examples of such risk stratification have been published ^8^ but, in our view, should be kept under review, given the false negative rate will vary with the prevalence of infection in the population. It may be prudent to only test key workers shortly after symptom onset, when the sensitivity of the test is highest.

It is biologically plausible that staff with SARS-CoV-2 infection may be less infectious if they have a negative PCR. We know of no good data to support such an assertion and would urge caution, as negative results can be a result of poor sampling or virus being less readily detected in the upper respiratory tract than in samples taken from the lower airways ^27^.

As others have also argued ^3^, we feel it likely that any gains in terms of reduced absenteeism as a result of a programme of testing symptomatic staff may be modest. Staff with symptoms may be too unwell to work and, regardless, should not be working whilst symptomatic given other respiratory viruses can also be transmitted to patients. Testing symptomatic household contacts may be more impactful with, potentially, a lower pre-test probability, allowing greater confidence in a negative result ^3^.

## Testing asymptomatic staff

Arguably, a better use of tests, particularly if capacity is limited, would be systematic regular testing of asymptomatic staff, to drive down the prevalence of asymptomatic and pre-symptomatic infection within institutions caring for the vulnerable. One model suggests that weekly PCR testing of asymptomatic health and social care workers could reduce their contribution to incident infections by 25–33%, over and above any reductions as a result of them self-isolating at onset of symptoms ^3^. Given virus is likely only detectable 1–2 days prior to symptom onset ^13, 23^, one might expect more frequent testing to result in greater declines in transmission, particularly if results could be fed back promptly. A second model gave similar results regards the impact of weekly testing and suggested that daily testing of hospital staff could reduce healthcare worker to healthcare worker transmission by 65% and healthcare worker to patient transmission by 14% ^7^.

Whilst, initially, a programme of testing asymptomatic staff might result in additional absenteeism, reductions in transmission may result in a favourable impact on staffing levels in the longer term ^7^. False positive tests would cause unnecessary absence from work, but no direct harm to vulnerable individuals.

The main barrier to regular asymptomatic testing is test capacity. For example, there are 1.5 million people working in adult social care in England ^28^. Pooling samples prior to testing could make regular systematic large scale testing of key workers feasible. This is discussed in detail below.

The impact of asymptomatic staff testing could be increased by calibrating test frequency to the proportion of staff and patients testing positive and by offering enhanced support with other aspects of IPC to facilities with higher than expected rates of positive tests – this approach has been described in the hospital setting ^8^.

## Testing asymptomatic patients

Following reports of high rates of asymptomatic infection in patients presenting to hospital with other issues ^18^, many hospitals are now undertaking SARS-CoV-2 PCR tests on all new admissions, with the result used to distribute patients to ‘COVID areas’, ‘non COVID areas’ or side rooms. With potential for harm should people with SARS-CoV-2 infection and vulnerable people who have not yet been infected be placed in the same space, it is important to think critically about the positive and negative predictive value of the test in specific circumstances.

People with a positive PCR result and symptoms consistent with COVID-19, as well as asymptomatic individuals with a negative PCR test, pose fewer challenges when drafting infection control guidelines. Note, however, that the latter group may subsequently become PCR positive, either a result of nosocomial infection or because they were in their incubation period and PCR negative at presentation.

People with COVID-19 symptoms, or imaging and blood tests consistent with COVID, but a negative SARS-CoV-2 PCR are commonly seen in clinical practice ^29^. Sometimes repeat PCR testing, particularly if a sample from the lower respiratory tract can be obtained, can confirm the diagnosis. Efforts should also be made to exclude alternative diagnoses, such as *Pneumocystis jirovecii* pneumonia, which can present in a similar manner.

The opposite phenomenon – a positive SARS-CoV-2 PCR in an asymptomatic person – can also occur. However, the possibility of this being an erroneous result is less well appreciated. In Table 2, are the probabilities of SARS-CoV-2 infection given a positive PCR that might be expected under various scenarios. False positive results will commonly be seen, particularly in settings and populations where the prevalence of infection is low.

**Table 2.** Estimated probability of SARS-CoV-2 infection given a positive PCR under various assumptions about the true prevalence of infection and the sensitivity of the test ^24^. All estimates assume a test specificity of 99.5%.

True prevalence of infection	Probability of SARS-CoV-2 infection, given positive PCR test		
	95% test sensitivity	70% test sensitivity	40% test sensitivity
15%	97%	96%	93%
5%	91%	88%	81%
1%	66%	59%	45%

Ideally, individuals with PCR test results that are not concordant with their symptoms would be cared for in side rooms. However, many institutions have a limited number of side rooms, meaning such a policy may not be sustainable. Requiring a second positive test in asymptomatic people with an initial positive PCR would seem prudent before moving them into a ‘COVID area’.

Where individuals’ infection status cannot be resolved and side rooms are not available, every effort should be taken to avoid placing individuals who would be particularly at risk of severe disease should they newly acquire infection in a space with individuals who may be infectious. Such a triage approach has been piloted at one hospital – in this case with the triage decision taken prior to the PCR result being available ^29^. The performance of any such algorithm will vary with the background prevalence of SARS-CoV-2 infection. If vulnerable and potentially uninfected individuals must be moved out of side rooms, impeccable infection control measures should be adopted in the spaces to which they are moved, perhaps supported by better staff-to-client ratios. If possible, such spaces should be closed to new admissions to prevent these individuals being exposed to new people who may be infectious.

## Pooling specimens prior to analysis

One way that testing capacity might be expanded is by pooling specimens prior to analysis. A recent report suggests that pooling of samples either prior to or following RNA extraction can allow multiple samples to be tested in a single PCR reaction without significant loss of sensitivity ^30^.

The authors reported a 1.24 increase in the PCR cycle threshold with every two-fold dilution of the sample – i.e. going from one to two, or two to four samples. In their hands, pooling 32 samples following RNA extraction reduced the sensitivity of the test by 10%. They suggest that some of this loss of sensitivity might be overcome by running the PCR for a few additional cycles. Any loss of specificity as a result of additional PCR cycles would not be expected to impact patient management, as individual samples from pools that tested positive would then be retested using one PCR reaction per sample.

The probability that, in any pool of specimens, at least one will test positive is one minus the probability that all will test negative. Here, the prevalence is the prevalence of positive tests, were each to be tested in a separate PCR run, rather than the true prevalence of infection.

*Probability ≥1 positive = 1 – (1 – prevalence) ^ number of tests*

The expected number of pools that test positive would be the product of this probability, the number of pools, and the sensitivity of the pooled approach, as compared with one PCR per sample. The number of infections missed, as compared with a one PCR reaction per sample strategy, would approximate the product of the true prevalence of infection, the loss of sensitivity as compared with a one PCR reaction per sample strategy, and the number of individuals tested.

**Figure 1.**
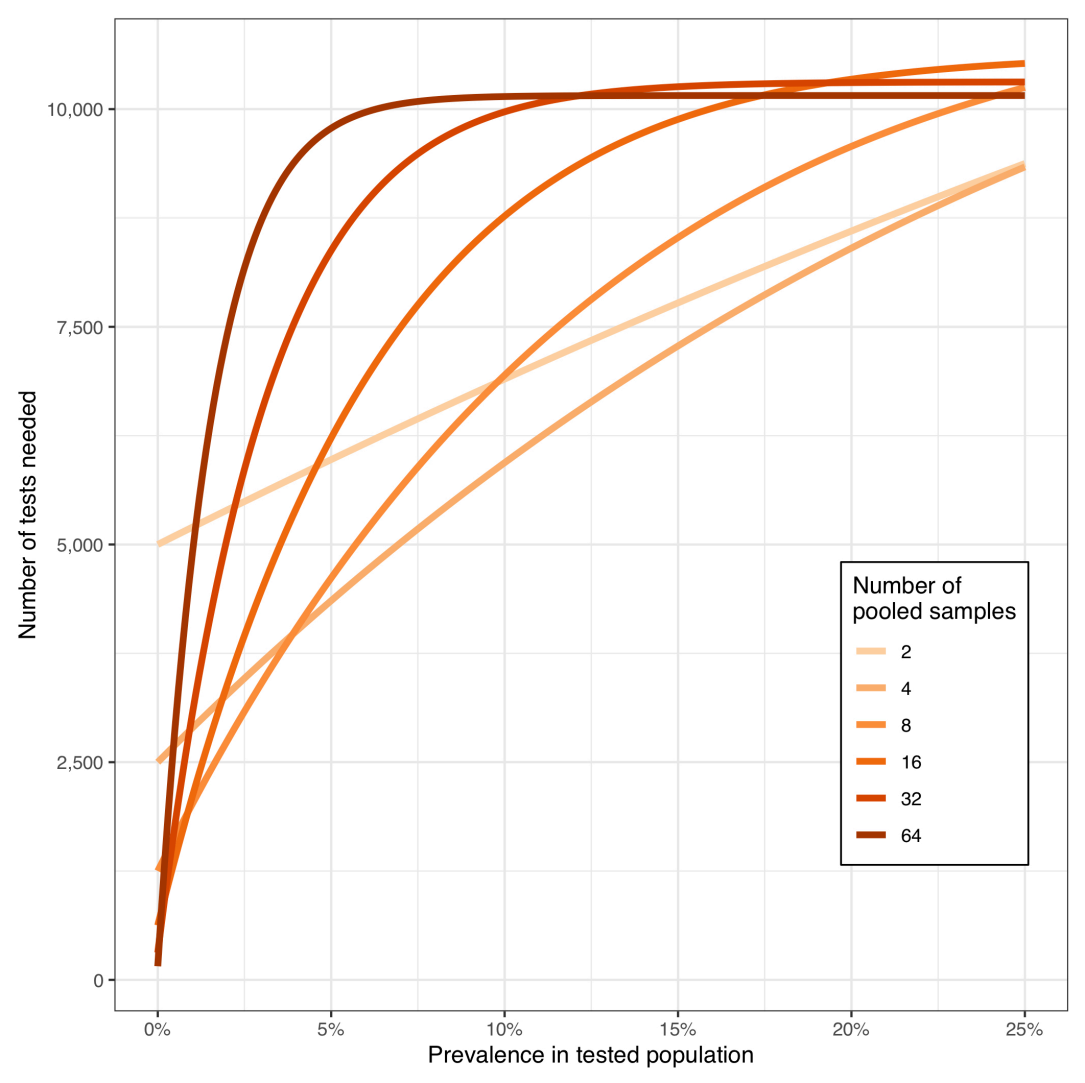
Number of PCR tests required as a function of pool size at different prevalences in a hypothetical population of 10,000 individuals.

The most efficient pool size depends on the prevalence ( Figure 1). The scenario illustrated in this figure assumes a health or social care workforce of 10,000 individuals and the number of PCR reactions calculated assumes individual PCRs are then performed on each sample in pools that test positive. Again, prevalence is the prevalence of positive tests, were each to be tested in a separate PCR run, rather than the true prevalence of infection.

The most efficient pool size is readily calculable, and the prevalence of positive tests should be monitored to ensure the optimal pool size is being used. At higher prevalence, larger pools are less efficient, as the high proportion of pools testing positive limits the number of tests saved.

Box 1. **Template staff testing strategy for institutions caring for vulnerable people**

- Briefing for staff on the planned testing strategy

- Sampling frame drawn up for each ward/facility, to include all medical, nursing, allied health professionals, and domestic staff with a regular presence in the institution

- Collect mobile phone numbers for each staff member, and issue each with a patient number, if they do not already have one

- Collate rotas and prepare two swabs for each staff member per week, pre-labelled and in lysis buffer (likely to equate to approximately alternate day testing, assuming some rest days)

- Allocate these to packets that are delivered to wards/facilities ahead of time with one packet for each morning handover

- After morning handover, self nose and throat swabs by outgoing night staff and incoming day staff

- Testing register to include space to note whether any staff have left or joined – swabs for new staff could be added to subsequent test packets

- Specimens collected after handover to allow RT-PCR for SARS-CoV-2 on a mid morning PCR run – this may need a dedicated courier collection

- Positive results called out before 5pm, to ensure incoming night staff don’t come on shift (if unable to contact staff member, message to be left with nurse in charge)

- Individual letters, emails or text messages to each staff member, containing test results, plus reliable and timely communication of positive results to local infection control and health protection teams

- Clear messaging to ensure symptomatic staff do not wait for their test result before leaving work, and that individuals developing symptoms self isolate regardless of whether they have had a recent negative test

- Dedicated mobile phone and email address for any queries

## Organisational considerations for asymptomatic staff testing

In designing a testing strategy for asymptomatic staff, there will be trade offs. For example, between a one PCR reaction per sample strategy and a pooled testing strategy. The former would be expected to result in the fastest results if there was plenty of capacity, whereas the latter would make most efficient use of limited capacity but add a few hours to the turn around time.

Duration of infectiousness prior to symptom onset is thought to be between 2–3 days ^13^ with limited PCR sensitivity expected more than two days prior to symptom onset ^23^. We would, therefore, advocate an asymptomatic staff testing strategy that prioritises frequent testing and prompt reporting of results over marginal improvements in test sensitivity.

Having nose and throat swabs taken by individuals trained to do this might result in better sampling, increasing test sensitivity, but would place limits on how quickly testing could be scaled up. Of note, 1533 swabs taken by symptomatic staff at a hospital in England were all positive for RNAaseP, a human nucleic acid used as a sample quality control. This does not mean that sampling was perfect, but suggests that the swabs did all make contact with some human mucosa ^31^. In a smaller study, good agreement was seen between PCR results using self swabs and swabs taken by a trained professional ^32.^

There is some data to suggest that saliva may represent a viable alternative upper respiratory tract sample to nose and throat swabs for SARS-CoV-2 PCR. Of the two studies published to date, one found saliva had slightly better sensitivity than nose and throat swabs ^33^, and one found the opposite ^34^. As one might expect, given saliva samples are easier to collect, greater consistency was seen between PCR results on repeat saliva samples than with repeat nose and throat swabs ^33^. Where self-sampling is planned, laboratories may wish to validate PCR assays using saliva.

A suggestion as to how a mass testing strategy for staff might be organised is outlined in Box 1. Other choices may be more appropriate, depending on local circumstances. For example, care homes may wish to include regular visitors in their sampling frame and to mandate a recent negative PCR prior to visiting. Facilities using agency staff may wish to mandate a recent negative PCR prior to any shifts. Testing intensity could be calibrated to local epidemiology and testing capacity ^8^, with a move to daily testing when capacity is available or if high rates of asymptomatic infection or disease are seen within facilities. Testing programmes should be developed in close collaboration with clinicians, infection control teams, health protection teams and the workers that are to be tested. 

Duration of self-isolation should be compliant with local guidelines, which are likely to evolve as our understanding of duration of infectiousness changes. Unless staff are immunocompromised, mandating negative PCRs before return to work would, in our view, be over cautious given PCR tests may detect non viable virus. Immunocompromised people should probably not be undertaking patient facing duties currently. In the absence of good data on duration of immunity following natural infection, pragmatic decisions may need to be taken locally about whether individuals who have had a positive SARS-CoV-2 PCR continue to be included in asymptomatic staff testing programmes.

## Serological tests

If and when we develop an understanding of the immunological correlates of protection against SARS-CoV-2 infection and or COVID-19, and have validated assays to measure this, they may compliment PCR based testing. For example, if we had a serological assay that could identify individuals with protective immunity to infection or disease, positive serology could allow someone to be safely moved out of a side room and into a ‘COVID area’. Such a test could also identify individuals that would no longer need to be included in asymptomatic staff testing programmes. For both of these purposes, a highly specific test would be needed.

## Key messages

- Correct interpretation of SARS-CoV-2 PCR tests depends on the sensitivity of the test and prevalence of infection in the population being tested.
- Test sensitivity declines with time since onset of symptoms.
- The prevalence of infection will vary between groups, with the symptoms people report, and over the course of the pandemic – monitoring the proportion of PCR tests that are positive can give an estimate of the prior probability of a positive test.
- Where prevalence of infection is high, or more than three days have elapsed since symptom onset, a negative PCR may not reliably exclude SARS-CoV-2 infection and, in this situation, symptomatic individuals should isolate whatever their PCR result.
- Where the prevalence of infection is low, false positive PCR tests may be commonly seen – it is prudent to obtain a second positive test before placing vulnerable individuals with a single positive test in a space containing other people with SARS-CoV-2 infection.
- Using test capacity to reduce the prevalence of SARS-CoV-2 infection in institutions accommodating vulnerable individual should be a priority, with models predicting a substantial impact on transmission.
- A significant proportion of SARS-CoV-2 transmission occurs prior to symptom onset and there is a window of 1–2 days prior to symptom onset when a PCR test might be positive – regular testing of asymptomatic individuals and the prompt reporting of results are therefore needed.
- Test capacity can be substantially increased by pooling samples prior to testing – the most efficient pool size will depend on the prevalence of infection and is readily calculated.
- Physical distancing and meticulous infection prevention and control will remain essential in institutions caring for vulnerable people.

## Data availability

No data are associated with this article.
