# Supplementary material for: Rational use of SARS-CoV-2 polymerase chain reaction tests within institutions caring for the vulnerable
Source: F1000Res. 2020 Jul 2;9:671. [Version 1] doi: 10.12688/f1000research.24872.1 (PMC7489272; doi:10.12688/f1000research.24872.1)
Supplement: Peer review history

# Peer review: Reviewer response for version 1

**DOI:** 10.5256/f1000research.27443.r69772

**Published:** 2020-09-11

**Authors:** Katie Jeffery, Sheila Lumley

**Affiliations:** 1 Nuffield Department of Medicine, University of Oxford, Oxford, UK; 2 Oxford University Hospital NHS Foundation Trust, Oxford, UK; 3 Department of Infectious Diseases and Microbiology, John Radcliffe Hospital, Oxford University Hospitals NHS Foundation Trust, Oxford, UK

Yates *et al.* discuss the current and highly topical issue of infection prevention control in institutional care facilities, focusing on the benefits of high frequency surveillance of asymptomatic and pre-symptomatic individuals, on absenteeism and infection prevention control. The article covers a range of important considerations, including sampling strategies (self-swabbing and saliva collection), testing strategies in particular pooling of samples, result interpretation given different population prevalence, and organisational considerations for implementing such a strategy. This article discusses how the pooling of samples might overcome the current capacity issues when implementing a high frequency mass screening strategy, given current constraints with NHS testing capacity to deliver screening programmes for large numbers of staff or patients.

 A number of papers on screening programmes in healthcare workers show high rates of nosocomial acquisition in staff who were ‘COVID-facing’. Much of this occurred in the early part of the pandemic in the UK before the introduction of universal PPE, with individuals looking after elderly patients or those with atypical COVID-19 presentations in the early part of the pandemic, leading to mode of acquisition in line with the reference quoted (89% of infections in healthcare workers may be a result of nosocomial transmission). However, following the introduction of universal PPE, the literature also documents high rates of staff to staff transmission in non-patient facing areas likely due to a failure of staff to socially distance away from clinical areas. In a study of over 10000 healthcare workers in an acute Trust the greatest risk of staff infection was associated with having a Covid-19 infected contact in the household (eLife 2020;9:e60675 DOI: 10.7554/eLife.60675) ^1^. Most Healthcare settings are now seeing lower rates of nosocomial and staff to staff transmission, given universal mask wearing in the UK. The authors’ intended audience is those designing testing strategies in health and social care settings, however, a number of the strategies discussed also have broader application for mass screening in lower prevalence settings such as workplaces and educational facilities. Expanding local/regional access to testing, testing capacity, improving turnaround time, maintaining analytic performance appropriate for the setting and population prevalence will contribute importantly to the current national effort to curb the pandemic.

 Comments to address:

 Turnaround times – a key factor in high frequency testing, including pre-shift testing, is short turnaround times. How does a pooling strategy effect this – it seems unlikely that the necessary turnaround times (i.e. results before returning for the next shift) could be met if samples are being analysed in a laboratory setting and repeat testing of individual samples from a positive pool is required. See: https://www.ncbi.nlm.nih.gov/pmc/articles/PMC7325181/. ^2^ Other assay formats could be considered such as point-of-care nucleic acid LAMP and rapid antigen tests – but given reduced sensitivity in comparison with laboratory based PCR assays modelling with respect to population prevalence would need to be carefully considered.

 Referencing: The COVID literature is expanding at an exponential rate. As with much of current COVID literature, many papers already exist on similar topics, the context of this opinion article within the current literature isn’t clear in places, with some citations lacking. This article would benefit from a more thorough literature review in the following areas:

1. Sample pooling is already used in a number of settings, with CDC guidance on the topic ( https://www.cdc.gov/coronavirus/2019-ncov/lab/pooling-procedures.html). The widespread use isn’t represented in this paper, with only 1 reference. There are a number of other papers discussing pooling samples, more accurate representation of current literature is needed.
2. Testing of saliva – there are multiple recent papers on this topic.

 

 Please clarify this paragraph: '…high rates of false negative tests are seen when the prevalence of infection is high, and when the sensitivity of the test is low. The proportion of health and social care workers who have positive test results should be monitored with these data, ideally, disaggregated by symptoms. Where this proportion is high, there will be an unacceptable false negative rate and institutions should consider asking symptomatic staff to self isolate regardless of their test result.’ – when what proportion is high? The proportion of symptomatic vs asymptomatic? Proportion testing positive?

 Serology: “Such a test could also identify individuals that would no longer need to be included in asymptomatic staff testing programmes” – this assumes that we know whether these individuals can be re-infected and still shed/transmit virus. The SIREN project (SARS-CoV-2 Immunity & REinfection EvaluatioN) includes those previously infected so we can gain a better understanding of what having evidence of previous exposure means with respect to re-infection.

 The article would benefit from a discussion of the information technology constraints – these are huge. Patient/sample identification at the point of test, sample labelling with barcodes allowing easier run-through processes in diagnostic laboratories, IT to support pooling/separation of pools, result notification to allow responses on an individual level and for contact tracing. 

 Conclusion

 At the time of writing this review (September 2020) the UK government is promising mass coronavirus testing (‘operation Moonshot’) but the technology to deliver this does not yet exist. It is vitally important to consider how mass-surveillance testing could be rolled out in institutional care settings. The pace at which testing is required to move forwards is unprecedented compared with anything we have seen before, and this opinion piece with its careful consideration of testing utility at different levels of population prevalence and assay sensitivity will be a useful article.

Is the topic of the opinion article discussed accurately in the context of the current literature?

Partly

Are arguments sufficiently supported by evidence from the published literature?

Partly

Are all factual statements correct and adequately supported by citations?

Yes

Are the conclusions drawn balanced and justified on the basis of the presented arguments?

Yes

Reviewer Expertise:

COVID: Asymptomatic healthcare worker surveillance/testing/contact tracing, laboratory testing, assay evaluation, infection prevention and control

We confirm that we have read this submission and believe that we have an appropriate level of expertise to confirm that it is of an acceptable scientific standard.

## References

[1] : Differential occupational risks to healthcare workers from SARS-CoV-2 observed during a prospective observational study. *Elife*.2020;9: 10.7554/eLife.60675 10.7554/eLife.60675 32820721PMC7486122

[2] : Test sensitivity is secondary to frequency and turnaround time for COVID-19 surveillance. *medRxiv*.2020; 10.1101/2020.06.22.20136309 10.1101/2020.06.22.20136309

# Peer review: Reviewer response for version 1

**DOI:** 10.5256/f1000research.27443.r66235

**Published:** 2020-07-20

**Authors:** Charles Agoti

**Affiliations:** 1 Epidemiology and Demography Department, Kenya Medical Research Institute, Kilifi, Kenya; 2 Department of Public Health, School of Health and Human Sciences, Pwani University, Kilifi, Kenya

Yates *et al.* vividly discuss utility and inherent limitations of current PCR testing of SARS-CoV-2 in institutions caring for the elderly and vulnerable adults. They begin by summarizing the SARS-CoV-2 epidemiology in this key population. The laboratory testing practical considerations and implications they highlight are very useful for both their specific target audience and the general scientific community to keep vulnerable populations safe. The messages of importance of quick sample-to-answer turnaround time, frequent testing to identify asymptomatic and pre-symptomatic infections, that PCR is not 100% accurate and that testing resources can be conserved by pooled testing are very important in these times. They provide suggestions on optimizing staff testing in care homes to monitor any SARS-CoV-2 infection introduction using available resources sparingly. I have three minor suggestions for the authors to consider.

1. Comment on if PCR cycle threshold (Ct) value is a useful indicator in evaluating the infectiousness of both staff and those under care and how this can be incorporated in making decisions in a care facility and in recognizing potential false positives?
2. Comment on whether sensitivity and specificity differ between PCR assays and how this can impact SARS-CoV-2 results in care homes. Any recommendations here? There are hundreds of commercial PCR assays available for SARS-CoV-2 testing. Some of the available PCR test check for multiple SARS-CoV-2 genes concurrently and include an internal control.
3. Comment on how/whether PCR tests can be used to identify contamination in care homes e.g. environmental swabbing, wastewater/sewage monitoring. Is this a viable strategy to monitor introduction or ongoing shedding/transmission?

Is the topic of the opinion article discussed accurately in the context of the current literature?

Yes

Are arguments sufficiently supported by evidence from the published literature?

Yes

Are all factual statements correct and adequately supported by citations?

Yes

Are the conclusions drawn balanced and justified on the basis of the presented arguments?

Yes

Reviewer Expertise:

Molecular diagnostics/viral genomics/Respiratory/Enteric virus transmission in community settings.

I confirm that I have read this submission and believe that I have an appropriate level of expertise to confirm that it is of an acceptable scientific standard.
